# Medical Men in Congress

**Published:** 1850-01

**Authors:** 


*Medical Men in Congress.*—G. N. Fitch, M. D., late professor of Principles and Practice of Medicine in Rush Medical College, has been elected to Congress from the Northern District in Indiana. Aside from giving any expression of a political partizan character, we believe he will be an able and efficient representative. We hope he may by efforts in procuring a law against patenting nostrums, even more highly distinguish himself, than has Dr. Edwards, of Ohio, by the Drug Inspection Law. It is high time the national government had withdrawn its patronage from the quackery of patenting medical compounds and systems of practice.—*North Western Med. and Surg. Journal.*

